# Visual acuity outcome and complications of cataract surgery in Nigeria: a systematic review and meta-analysis

**DOI:** 10.1093/inthealth/ihaf161

**Published:** 2026-03-25

**Authors:** Saudat Garba Habib, Abdullahi Idris, Fatimah Isma’il Tsiga-Ahmed, Clare Gilbert

**Affiliations:** Department of Ophthalmology, Bayero University/Aminu Kano Teaching Hospital, PMB 3452, Kano, Kano State, Nigeria; Department of Ophthalmology, Rasheed Shekoni Federal University Teaching Hospital, Dutse, Jigawa State, Nigeria; Department of Community Medicine, Bayero University/Aminu Kano Teaching Hospital, PMB 3452, Kano, Kano State, Nigeria; International Centre for Eye Health, Department of Clinical Research, London School of Hygiene &Tropical Medicine, WC 1E 7HT Keppel Street, London, UK

## Abstract

This study estimated the pooled prevalence of visual acuity (VA) outcomes and potentially blinding cataract surgery complications in Nigeria. Ethical clearance was waived by the Ethical Review Board of the Kano State Ministry of Health. We searched PubMed, African journals online, Embase, MEDLINE and Google Scholar to identify relevant studies from January 1990 to February 2025. Data on presenting and best-corrected visual acuities at ≥6 weeks after cataract surgery, intraoperative posterior capsular rupture with vitreous loss and postoperative endophthalmitis were extracted from included studies. I^2^ statistics were used to assess heterogeneity across studies. Articles were systematically reviewed and a random effects meta-analysis model was applied to estimate the pooled effect size across studies. All statistical analyses were performed using Stata version 17.0 software. Sixteen studies, with a total of 3631 cataract-operated eyes, were included in the review. A total of 80.0% of all operated eyes had a preoperative VA of <3/60. At ≥4 weeks the percentage with good presenting VA ranged from 8% to 86%, with evidence of improved outcomes over time (95% confidence interval [CI] 45.0 to 67.0, I^2^=96.7%). With the best correction, 75% (95% CI 66.0 to 83.0, I^2^=98%) had a good outcome at ≥6 weeks postoperatively. The pooled prevalence of posterior capsular ruptures with vitreous loss was 4.0%, while 1.0% had postoperative endophthalmitis. In this review, the VA outcome after cataract surgery in Nigeria is well below the World Health Organization–recommended benchmark of >90% for a good result, with a higher rate of potentially blinding complications. The findings of this review suggest the need for improvement in modern surgical techniques, quality biometry and stocking of intraocular lenses of different powers and types to improve visual outcome and reduce complication rates.

## Introduction

Cataract is the most common cause of avoidable blindness worldwide,^1^ accounting for approximately 70% of treatable blindness and 34% of treatable moderate and severe visual impairment.^2^ The only effective treatment is surgical extraction, and cataract surgery is highly cost-effective, resulting in almost immediate visual restoration.^3^ Advances in cataract surgical techniques and the use of appropriate intraocular lenses (IOLs) after power calculation have resulted in better visual outcomes, with fewer complications and less postoperative morbidity.^4^ Poor surgical outcomes affect surgical services and coverages, especially in low-income settings where the burden of cataract blindness is high.^5^ Visual acuity (VA) outcomes following cataract surgery are important measures of assessing quality and patient safety. Monitoring outcomes with corrective action and appropriate surgical techniques can help improve services, particularly in high-volume centres.^6^ The effectiveness of case selection and the quality of the surgery, including the surgeon’s expertise to minimise the risk of complications, use of appropriate modern techniques, power of IOLs and postoperative management, influences the outcome of cataract surgery and decreases potential loss of vision in individuals.^7^

Posterior capsular rupture (PCR) with or without vitreous loss (VL) as well as endophthalmitis are the most common potential complications, resulting in vision loss,^8^ and reductions of these complications reduces healthcare costs.^9^ For instance, the UK’s national audit of cataract surgery reported a downward rate of postoperative VA loss, from 0.9% in 2010 to 0.51% in 2020. This has resulted in annual savings of £2.0 million from avoided additional treatment.^10^ The rate of these complications is widely regarded as the benchmark for measuring the quality of cataract surgery.^11^

The World Health Organization (WHO) has categorised outcomes of cataract surgery in the operated eye measured within a specific time of surgery as good (≥6/18), borderline (<6/18–6/60) and poor (<6/60).^12,13^ The WHO recommended achieving good uncorrected outcome in 80% of patients or >90% with best corrected VA (BCVA). These categories provide a benchmark, allowing comparisons to be made over time and between service providers.

Although different primary visual outcome studies have shown substantial benefits of visual restoration, variation in the VA outcomes of cataract surgery has been reported, particularly in low-income countries.^14^ In Nigeria, for example, in the national blindness prevalence survey, only 29.9% of the eyes of participants who had undergone cataract surgery had a good outcome (VA ≥6/18) using presenting VA (PVA), which increased to 55.9% with best correction,^15^ which is well below the WHO benchmark. This review of hospital-based studies of cataract surgery in Nigeria was conducted to describe pooled VA outcomes of cataract surgeries in adults using the WHO benchmark and the most common visually threatening complications (PCR with or without VL and endophthalmitis). This will aid in understanding the current outcome and suggest improvements.

## Methods

The Preferred Reporting Items for Systematic Reviews and Meta-analysis (PRISMA)^16^ guideline was used in this review. The protocol of this review was registered prospectively in PROSPERO (registration number CRD 42022354608).^17^

Ethical clearance was waived by the Ethical Review Board of the Kano State Ministry of Health. All hospital-based/audit-type cataract surgical outcome studies in Nigeria were considered in this review. The study included all cataract surgical techniques (i.e. extra capsular cataract extraction [ECCE], manual small incision cataract surgery [MSICS] or phacoemulsification with or without intraocular lens [IOL] implantation) and both retrospective and prospective studies were included.

### Search strategy

We conducted a systematic literature search of electronic databases of PubMed, Embase, MEDLINE and Google Scholar for published articles that reported visual outcomes after cataract surgery in Nigerian populations using the following search terms and keywords (formatted for PubMed search):(‘cataract surgery’ AND ‘outcome’ OR ‘visual outcome’ OR ‘hospital audit’ OR ‘hospital-based’ OR ‘facility-based’ ‘prospective studies’), (‘cataract’ [All Fields] OR ‘cataract surg*’ [All Fields] OR ‘outcome’[All Fields]), ‘Nigeria’ [Mesh] OR ‘Nigeri*’ [All Fields] OR ‘Nigeria’ [All Fields], (#1 OR #2) AND #3. References of accessed articles were also manually reviewed for additional articles and the corresponding authors of articles with restricted access were contacted to obtain the article.

### Eligibility criteria

Two reviewers (AI and SGH) independently screened titles and abstracts for relevance against the inclusion criteria. Articles that met any of the following criteria were included: standardized hospital-based audit of cataract surgery that reported VA outcomes with explicit categorization as good, moderate or poor using WHO categories; published in peer-reviewed journals or grey literature and published in English from January 1990 to February 2025.

The following studies were not included: population-based cross-sectional studies, studies including children of all ages where data were not disaggregated by age group, surgical procedure was not described, IOLs were not used, no sample size was reported WHO categories were not used and the data could not be extracted.

Duplicates were removed and only the full text of studies that met inclusion criteria were obtained and screened for methodological rigour and quality checks. A consensus was reached between the two reviewers in the case of discrepancies.

### Outcome measure

We defined the primary outcome as the prevalence of good (VA <6/18) PVA and BCVA at 4 and ≥6 weeks, respectively, after cataract surgery. The secondary outcomes were the pooled prevalence of eyes with PCR with or without VL during cataract surgery and postoperative endophthalmitis.

### Data extraction

Data were extracted independently using Excel forms (Microsoft, Redmond, WA, USA) developed specifically for this review. The data extracted from each article included the first author’s name; publication year; journal name; region; study design; data collection period; sample size; number of eyes undergoing cataract surgery; age (mean and range); surgical technique(s); preoperative VA; duration of follow-up; VA outcome categorized as good, borderline or poor over a specified period with and without correction; proportion of IOL use; complications associated with risks of visual loss such as PCR, VL and endophthalmitis, as well as reasons for poor outcomes. Data extracted were compared and assessed by both authors together and discrepancies were resolved by consensus and referring to the original article.

### Quality assessment of included studies

The methodological quality of each included study was assessed using the modified Newcastle–Ottawa scale.^18^ The scale is primarily formulated by a star allocation system, with a maximum of 10 stars across three domains. Studies that scored ≥5 stars in each of the three domains were included. Quality assurance checks were performed by two authors (AI and SGH) and discrepancies were resolved by consensus, presented in Supplementary Table S1.

### Risk of bias assessment

Hoy et al.^19^ developed a tool for assessment of internal and external validities in non-randomized studies in meta-analyses. The two authors carried out a risk of bias assessment using the Hoy scores and studies were classified based on the scores obtained into low (total score 8–10), moderate (total score 5–7) and high risk, which are presented in Supplementary Table S2.

### Heterogeneity and publication bias

Cochran’s Q and I^2^ statistics were employed to investigate heterogeneity between studies,^20^ which estimates the percentage of total variation across studies due to true between-study differences rather than chance, with I^2^ values of 25%, 50% and 75% representing low, medium and high heterogeneity, respectively. Sources of heterogeneity were explored through subgroup analysis and meta-regression analysis. The effect size of each study on the overall prevalence was also evaluated through sensitivity analysis for each study. Publication bias was assessed by visually inspecting funnel plots and objectively using Egger’s test.^21^

### Statistical analysis

We estimated the pooled proportion of preoperative VA, PVA and BCVA using the WHO cataract surgery outcome monitoring categorization for good, borderline and poor outcomes with meta-analysis weighting. All pooled proportions are presented using tables or forest plots. A random effects model^22^ was chosen due to the variability in the study populations, surgical techniques, age of participants and timing of measurement of the outcome. The proportion of total variation across studies due to heterogeneity was evaluated by the I^2^ measure. Statistical analyses were performed using the metaprop^23^ command within Stata version 17.0 IC (StataCorp, College Station, TX, USA),^24^ which presents pooled estimates with inverse variance weights. In pooling some outcomes with proportions close to or at 1, we enabled the Freeman–Turkey double arcsine transformation to prevent the exclusion of some studies.

## Results

**Figure 1.**
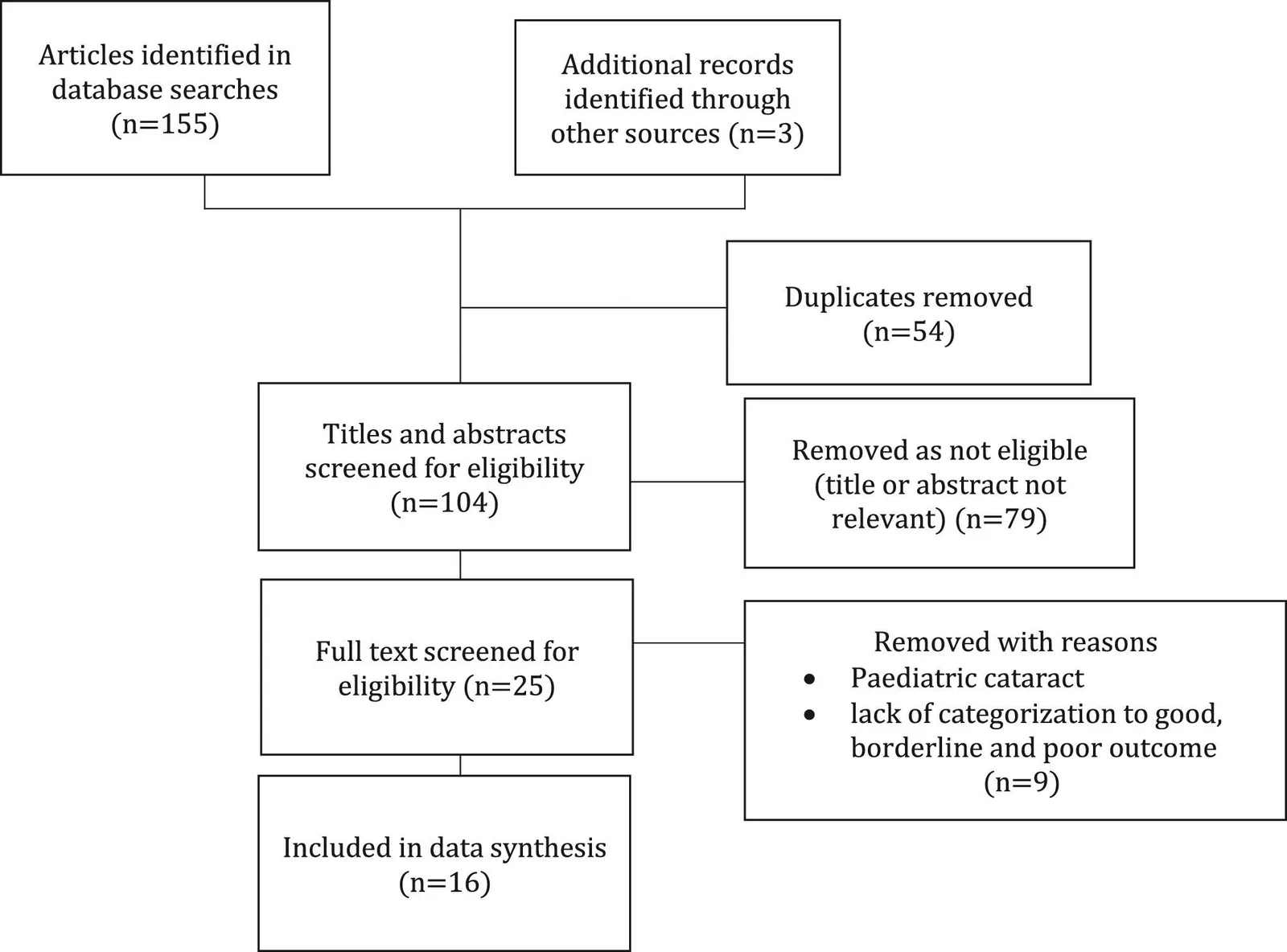
PRISMA flow diagram for study identification and selection process.

A total of 155 potentially relevant titles and abstracts were identified from the initial database searches and three records were identified from other sources. After removing 54 duplicates, we screened abstracts of 104 articles for relevance and 79 abstracts were rejected. The full text of 25 articles was obtained and reviewed for inclusion and quality assessment, 9 of which were excluded due to inadequate data on VA or data on all ages were not disaggregated. We included 16 articles that were published between 2000 and 2025 (Figure 1).

### Baseline characteristics of studies reporting VA outcomes

Sixteen studies with a total of 3631 eyes of 3491 patients were included in the quantitative analysis. Studies were undertaken in five of the six geographical zones in Nigeria, with the largest number in the southwest region, which is where the largest city (Lagos) is located. Participants were of both genders, ages ranging from 19 to 100 y, with a mean age of 54.2 y. The median follow-up time was 8 weeks (range 4–72, interquartile range 12 weeks).

The surgical techniques used were as follows: 6 (37.5%) studies reported ECCE with PCIOL, align="center" width="4pc"/ 4 (31.25%) used MSICS with PCIOL, 1 (6.25%) used phacoemulsification and 4 (25.0%) used a combination of two of the techniques (Table 1).

**Table 1.** Details of 16 included studies providing data on VA after cataract surgery.

										Outcome of cataract surgery						
						Pre-operative visual acuity (%)				Presenting VA (%)			BCVA (%)			
Author	Year of publication	Study design	Location (region)	Eyes, n	Surgical technique	<6/18–6/60	<6/60–3/60	<3/60	IOL used (%)	Good	B’line	Poor	Good	B’line	Poor	Quality score
Alhassan et al.^25^	2000	R	Kaduna(NW)	175	ECCE+IOL	10.7	3.5	85.8	100	45.2	50.6	4.2	54.6	33.3	12.1	8
Ezegwui and Ajewole^26^	2009	P	Akwa Ibom(SE)	100	ECCE+IOL	1.0	1.0	98	ND	8.1	48.9	42.8	35.4	50	14.6	7
Obiudu et al.^27^	2009	P	Imo(SE)	108	ECCE+IOL	ND	6.5	93.5	100	59	32	9.0	ND	ND	ND	5
Odugbo et al.^28^	2009	P	Plateau(NC)	219	ECCE+IOL	ND	ND	83.6	100	53.4	35.4	11.2	73.6	17.6	8.8	7
Olawoye et al.^29^	2010	P	Ibadan(SW)	146	SICS+IOL	4.1	19.2	75.3	100	37	50.7	12.3	79.5	13.0	7.5	8
Olawoye et al.^30^	2011	R	Ibadan(SW)	165	ECCE+IOL	7.1	17.4	74.5	100	38.8	47.3	13.9	74.6	20.6	4.8	8
Oladigbolu et al.^31^	2014	R	Kaduna(NW)	690	ECCE+IOL	ND	ND	71.2	100	53.6	ND	ND	ND	ND	ND	5
Ejinmadu and Pedro-Egbe^32^	2014	P	Rivers(SS)	92	SICS;ECCE+PCIOL	19.6	7.6	72.8	ND	ND	ND	ND	66.3	23.9	9.8	5
Nkanga et al.^33^	2015	P	Calabar(SS)	82	SICS+IOL	4.9	2.4	92.7	97.5	70.7	24.4	4.9	ND	ND	ND	6
Shu’aibu et al.^34^	2015	R	Kano(NW)	360	Ph/ECCE+IOL	ND	ND	ND	ND	79.0.	19.0	2.0	ND	ND	ND	5
Oderinlo et al.^35^	2017	R	Lagos(SW)	157	Ph+IOL	38.2	26.1	35.7	100	86	14	1.0	98	1.3	0.7	9
Fasina et al.^36^	2017	R	Ibadan(SW)	460	SICS/ECCE+IOL	3.3	6.5	90.2	97.8	57	26	17	72.9	16	11	9
Oladigbolu et al.^37^	2021	P	Kaduna(NW)	405	SICS+IOL	ND	ND	88.4	100	71.5	28.5	0.0	84.2	11.7	4.10	7
Bulus et al.^38^	2022	P	Kaduna (NW)	116	SICS+IOL	0.00	29.4	70.6	94.0	59.6	32.1	8.3	75.2	19.3	5.5	8
Bogunjoko et al.^39^	2023	R	Ogun(SW)	250	SICS,PHACO	30.4	2.0	60	ND	88	9.2	2.8	93.2	4.0	2.8	6
Jeneral et al.^40^	2023	R	Plateau(NC)	87	SICS+IOL	ND	ND	ND	100	36.8	47.1	16.1	78.1	13.8	8.1	5

R: retrospective; P: prospective; ND: no data; B’line: borderline.

ECCE+IOL: extracapsular cataract extraction with posterior intraocular lens; SICS+PCIOL: small incision cataract surgery with posterior intraocular lens; Ph: phacoemulsification.

### Preoperative VA

All included studies reported VA before surgery. Fourteen studies reported a VA of <3/60, with proportions ranging from 35.7% to 93.5%; the pooled proportion was 80.0% (95% CI 72.0 to 87.0), with high interstudy heterogeneity (I^2^=96.37%). Twelve studies reported preoperative VA of 6/60–3/60 (proportions ranging from 5.0% to 15.0%) with a pooled proportion of 9.0% (95% CI 5.0 to 15.0). Eleven studies reported a VA of <6/18–6/60, with proportions ranging from 1.0% to 38.2% and a pooled proportion of 9.0% (95% CI 3.0 to 16.0), as shown in Table 2.

**Table 2.** Pooled proportion of presenting pre-operative VA categories.

	Category of presenting preoperative VA					
	VA <3/60		VA <6/60–3/60		VA <6/18–6/60	
Study	Effect size	CI	Effect size	CI	Effect size	CI
Alhassan et al.^25^	0.86	0.80 to 0.90	0.04	0.02 to 0.07	0.11	0.07 to 0.16
Ezegwui and Ajewole^26^	0.98	0.92 to 0.99	0.01	0.00 to 0.05	0.01	0.00 to 0.05
Obuidu et al.^27^	0.94	0.87 to 0.97	0.06	0.03 to 0.13		
Odugbo et al.^28^	0.84	0.78 to 0.88	−0.12	0.09 to 0.17	−0.04	−0.02 to 0.08
Olawoye et al.^29^	0.75	0.68 to 0.82	0.19	0.14 to 0.26	0.04	0.02 to 0.09
Olawoye et al.^29^	0.75	0.68 to 0.80	0.17	0.14 to 0.26	0.07	0.04 to 0.12
Oladigbolu et al.^31^	0.71	0.68 to 0.74	–	–	–	–
Ejimadu and Pedro-Egbe^32^	0.73	0.63 to 0.81	0.08	0.04 to 0.15	0.20	0.13 to 0.29
Nkanga et al.^33^	0.93	0.85 to 0.97	0.02	0.01 to 0.08	0.05	0.02 to 0.12
Oderinlo et al.^35^	0.36	0.29 to 0.43	0.26	0.20 to 0.33	0.38	0.31 to 0.46
Fasina et al.^36^	0.90	0.87 to 0.93	0.06	0.05 to 0.09	0.03	0.02 to 0.05
Oladigbolu et al.^37^	0.88	0.85 to 0.91	–	–	–	–
Bulus et al.^38^	0.71	0.62 to 0.78	0.29	0.22 to 0.38	0.00	0.00 to 0.03
Bogunjoko et al.^39^	0.60	0.54 to 0.66	0.02	0.01 to 0.05	0.30	0.25 to 0.36
Random pooled effect size	0.8	0.72 to 0.87	0.09	0.05 to 0.15	0.09	0.03 to 0.16

### Postoperative VA

**Figure 2.**
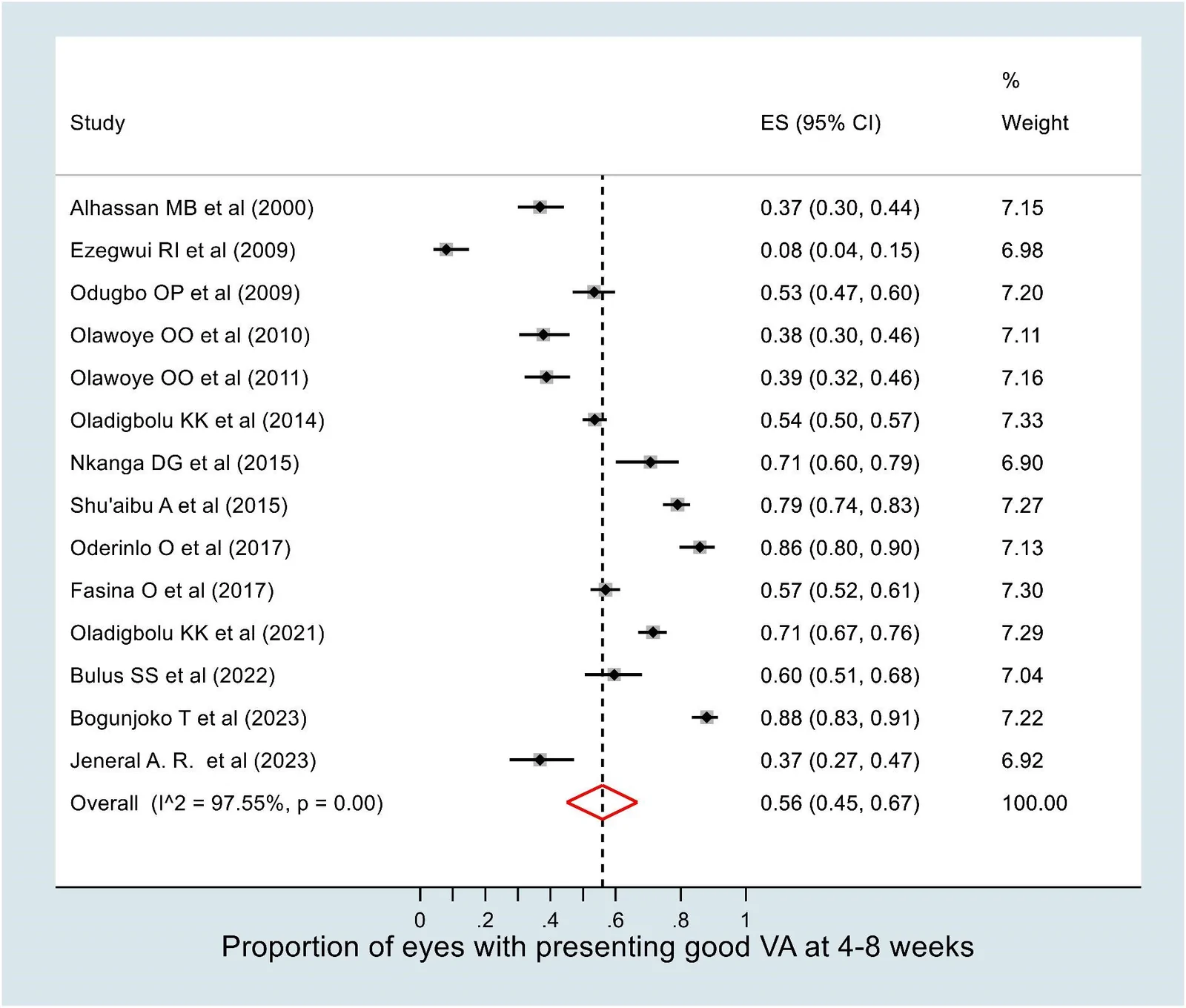
Forest plot of pooled estimates of eyes with good presenting VA at ≥4 weeks after surgery.

**Figure 3.**
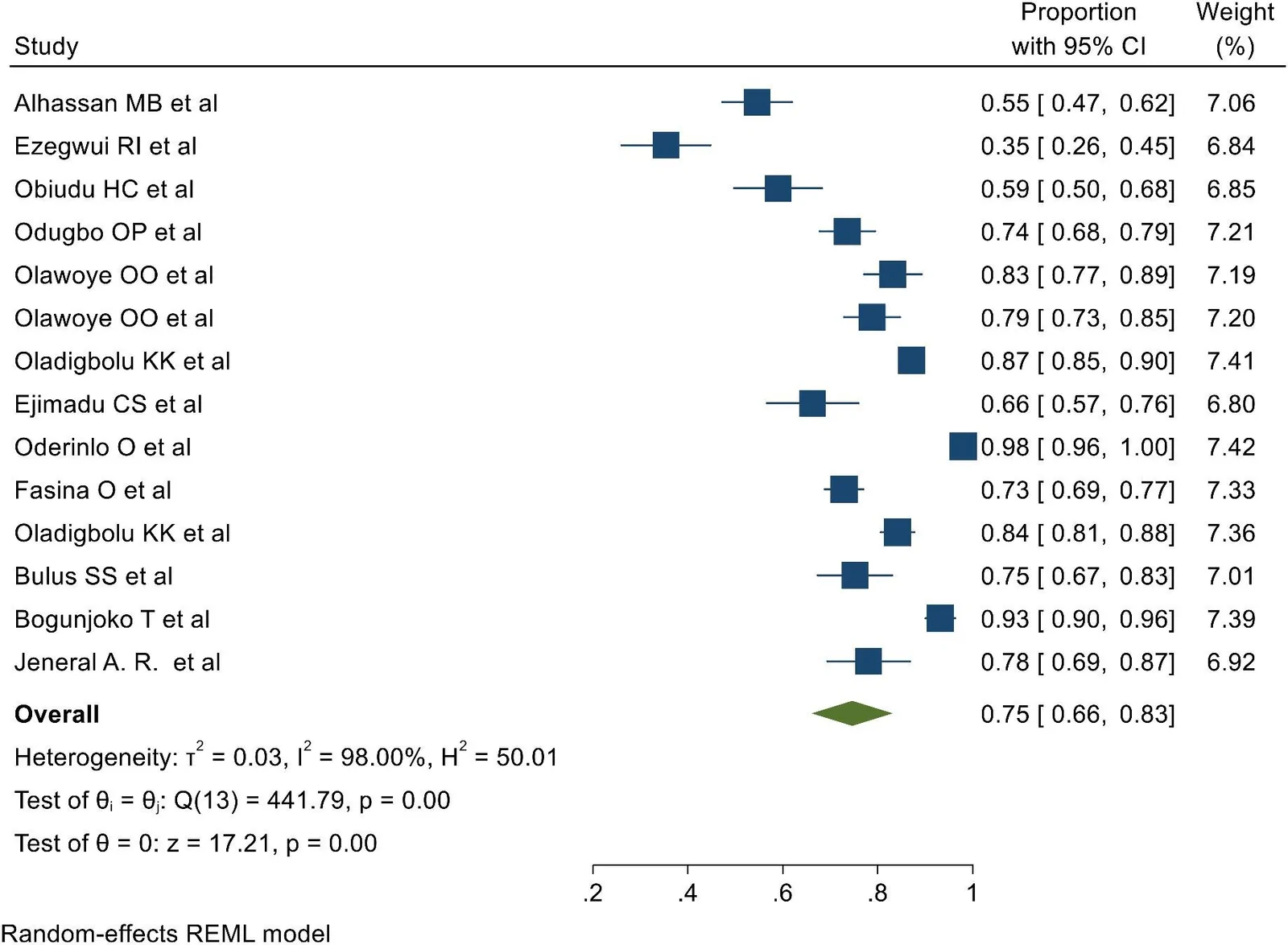
Forest plot of pooled estimates of eyes with good BCVA ≥6 weeks after surgery.

Fourteen studies reported data on good PVA at ≥4 weeks and 14 reported good BCVA at ≥6 weeks. The aggregated estimate was 56.0% (95% CI 45.0 to 67.0) for good PVA at ≥4 weeks (Figure 2), which increased to 75.0% (95% CI 66.0 to 83.0) with best correction at ≥6 weeks (Figure 3). There was significant heterogeneity between studies for good PVA outcomes (range 8–86%, heterogeneity χ^2^=298.00, p<0.001, I^2^=97.65) as well as for good BCVA outcomes (range 35–98%, T^2^=0.03, I^2^=98.0%, p<0.001).

Using WHO recommendations, the proportion of eyes with good PVA from this meta-analysis was much lower (56%) than the 80% recommended, however, the proportion with borderline (33%) and poor (8%) PVA were similar to the WHO standard. A different pattern was observed with BCVA, where the proportion of eyes with good BCVA (75%) was lower than WHO recommendations (90%) but the borderline category was higher (20%) (Table 3).

**Table 3.** Comparison of pooled prevalence of good, borderline and poor VA at >6 weeks postsurgery, before correction and WHO recommended.

	Presenting VA, %		BCVA, %	
VA categories	WHO benchmark	Nigeria	WHO benchmark	Nigeria
Good (≥6/18)	80	54	90	75
Borderline (6/18–6/60)	15	15	5	15
Poor (<6/60)	5	6	5	6

### Subgroup analysis

To identify the source of heterogeneity across the included studies, subgroup analysis was deployed using regions, study design, surgical techniques and duration of follow-up. Stratified by location, southwestern Nigeria had the highest prevalence at 85.0% (95% CI 76.0 to 95.0), followed by northwest at 76.0% (95% CI 68.1 to 90.0), northcentral at 75.0% (95% CI 70.0 to 80.0), southeast at 59% (95% CI 50.0 to 68.0) and south at 51% (95% CI 21.0 to 81.0). Furthermore, the pooled proportion of eyes with good VA 6 weeks after best correction was higher among studies with a retrospective design (80.0% [95% CI 67.0 to 92.0]) than those with a prospective design (71.0% [95% CI 59.0 to 83.0]) (Table 4).

**Table 4.** Results of subgroup analysis by different categories of the studies.

Subgroup	Categories	Studies, n	Sample size, n	Proportion with 95% CI	p-Value	I^2^, %
Region	Northwest	4	1048	76 (61 to 90)	0.00	97.59
	South	2	192	51 (21 to81)	0.00	95.06
	Southeast	1	108	59 (50 to68)	–	–
	Northcentral	2	306	75 (70 to80)	0.40	0.00
	Southwest	5	1193	85 (76 to95)	0.00	96.67
Study design	Retrospective	6	1313	80 (67 to92)	0.00	98.13
	Prospective	8	1867	71 (59 to83)	0.00	97.35
Surgical techniques	ECCE+PCIOL	6	1476	65 (50 to80)	0.00	97.54
	MSICS+PCIOL	4	690	81 (77 to85)	0.16	43.87
	MSICS+PCIOL, ECCE+PCIOL	2	552	71 (65 to77)	0.22	34.39
	PHACO+PCIOL	1	157	98 (96 to 100)	–	–
	MSCIS+PCIOL,PHACO+PCIOL	1	250	93 (90 to96)	–	–
Duration of follow up	≤6 weeks	5	1033	69 (61 to77)	0.00	85.85
	>6 weeks	9	2140	77 (65 to90)	0.00	98.83

### Meta-regression analysis

Using meta-regression, we observed an increase in the proportion of eyes with good VA at 6 weeks after best correction at a rate of 0.90% per additional eye studied (p<0.001). Although there was insufficient evidence, an increase of 1.9% in the proportion of eyes with good VA per year was also observed.

### Sensitivity

**Figure 4.**
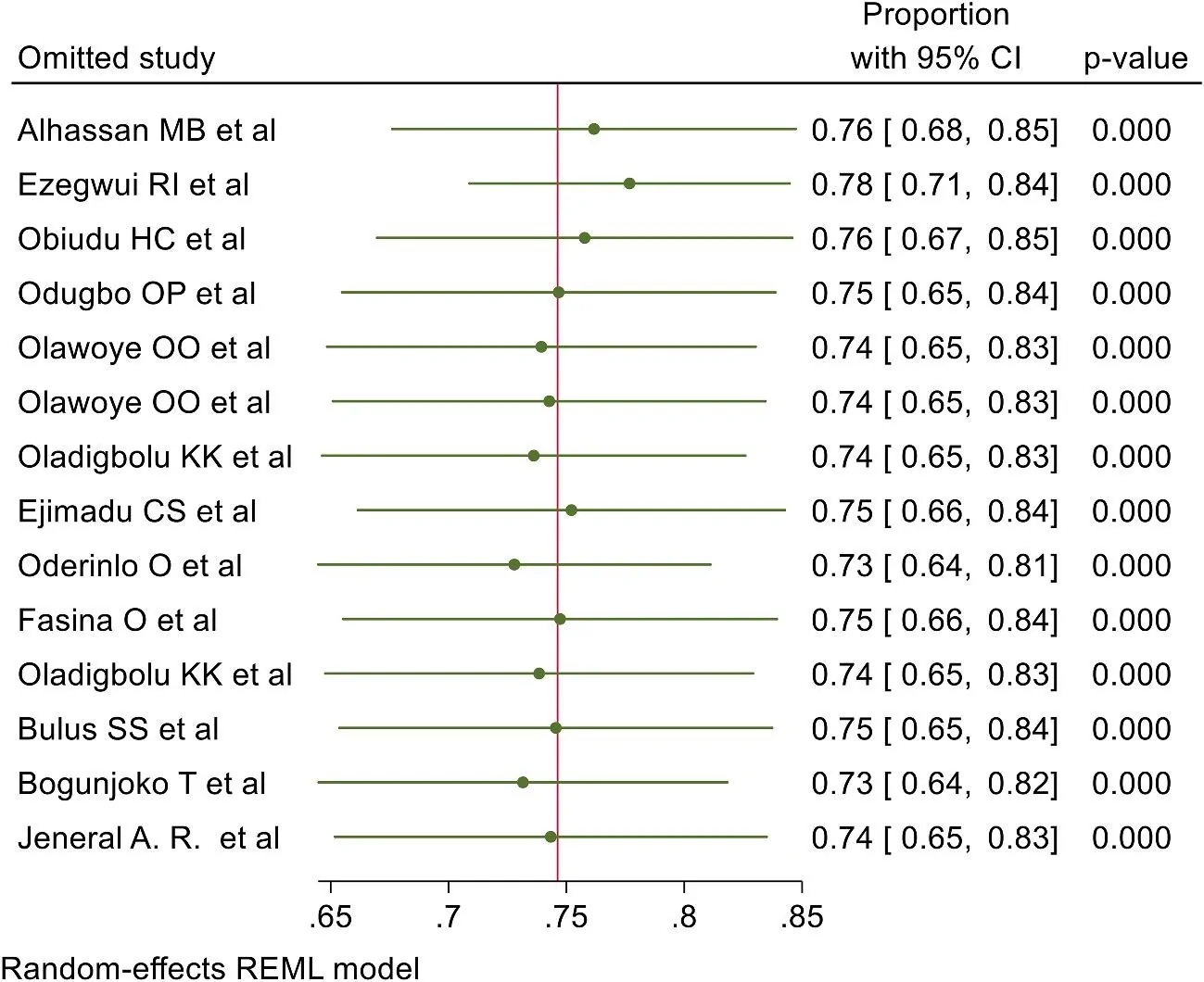
Results of the sensitivity analysis of the 14 studies.

The leave-one-out sensitivity analysis revealed that no single study significantly impacted the reported pooled proportion of eyes with good VA at 6 weeks after best correction. The pooled rates varied throughout the sequential leave-one-out analysis iterations, ranging from 73.0% (95% CI 64.0 to 82.0; p=0.000) to 78.0% (95% CI 71.0 to 84.0; p=0.000) (Figure 4).

### Publication bias

**Figure 5.**
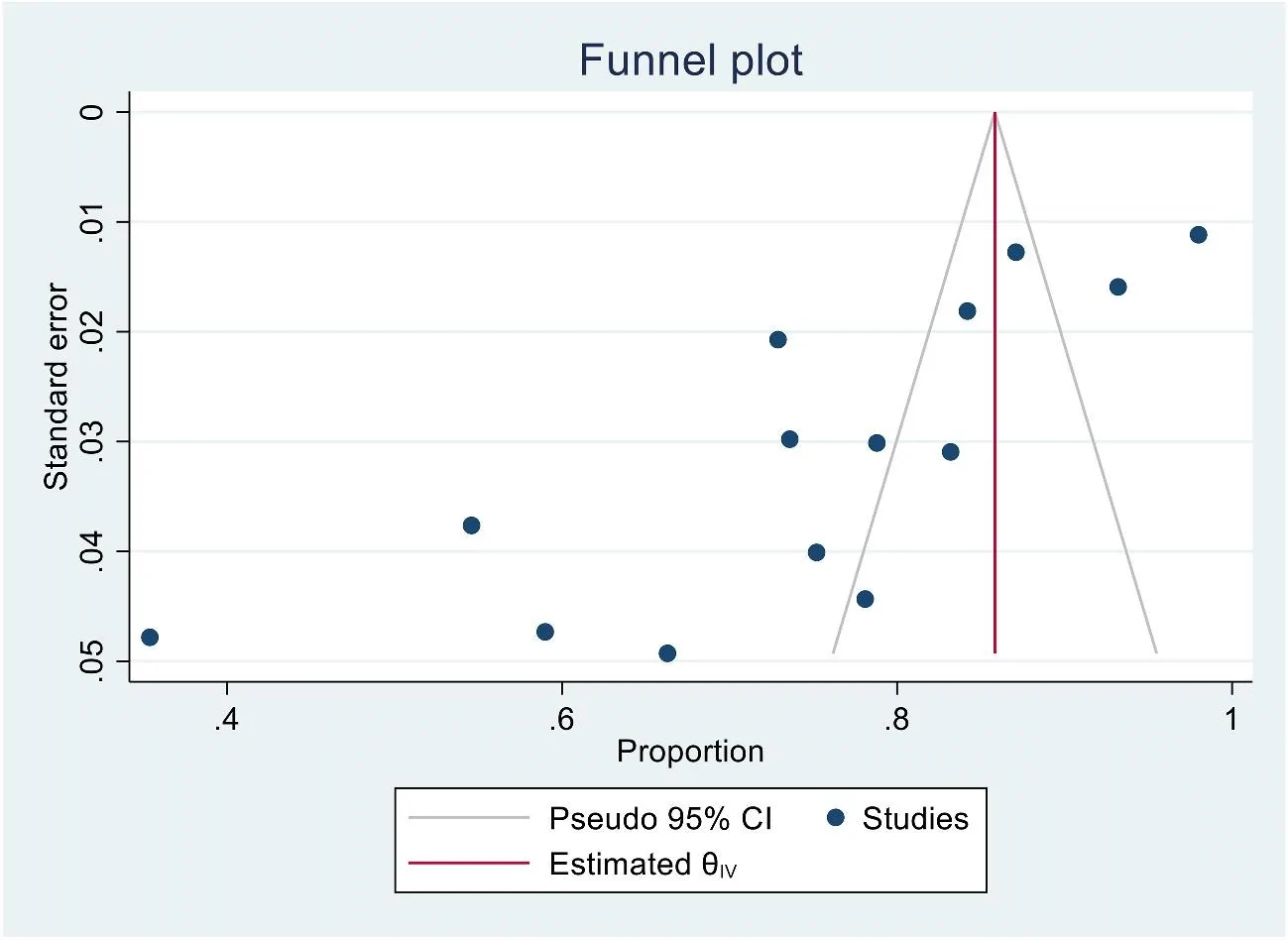
Funnel plot to test publication bias of the 14 studies.

Some evidence of publication bias was observed by visual inspection of Begg’s funnel plot and by using Egger’s test (p≤0.01) for included studies (Figure 5).

### Major complications

Thirteen studies (N=2052) reported intraoperative complications. PCR with VL ranged from 1.0% to 23.1%, with a pooled prevalence of 4% (95% CI 1.0 to 8.0). PCR without VL ranged from 2.0% to 23.0%, with a pooled prevalence of 7.0% (95% CI 5.0 to 10.0) (Table 5).

**Table 5.** Pooled proportion of PCR with VL (12 studies) and endophthalmitis (8 studies).

	PCR with VL		PCR without VL		Endophthalmitis	
Study	Effect size	95% CI	Effect size	95% CI	Effect size	95% CI
Alhassan et al.^25^	0.02	0.01 to 0.05	0.06	0.03 to 0.10	0.01	0.00 to 0.03
Ezegwui and Ajewole^26^	0.05	0.02 to 0.12	0.06	0.03 to 0.12	No data	No data
Obiudu et al.^27^	0.03	0.01 to 0.08	0.04	0.01 to 0.09	No data	No data
Odugbo et al.^28^	0.08	0.05 to 0.13	0.15	0.11 to 0.20	No data	No data
Olawoye et al.^29^	No data	No data	0.07	0.04 to 0.12	0.01	0.00 to 0.04
Olawoye et al.^29^	No data	No data	0.05	0.03 to 0.10	0.01	0.00 to 0.03
Oladigbolu et al.^31^	0.00	0.00 to 0.01	0.03	0.02 to 0.04	0.003	0.001 to 0.011
Ejimadu and Pedro-Egbe^32^	0.01	0.00 to 0.05	No data	No data	0.03	0.01 to 0.09
Nkanga et al.^33^	0.11	0.06 to 0.20	0.11	0.06 to 0.20	No data	No data
Shuaibu et al.^34^	0.00	0.00 to 0.01	No data	No data	0.00	0.00 to 0.01
Oderinlo et al.^35^	0.02	0.01 to 0.05	0.02	0.01 to 0.05	No data	No data
Fasina et al.^36^	0.09	0.06 to 0.12	0.10	0.08 to 0.14	0.01	0.00 to 0.02
Oladigbolu et al.^37^	0.05	0.03 to 0.07	0.05	0.03 to 0.07	No data	No data
Bulus et al.^38^	0.23	0.01 to 0.08	0.23	0.16 to 0.32	0.01	0.00 to 0.02
Random pooled effect size	0.04**	0.01 to 0.08	0.07	0.05 to 0.10	0.01	0.00 to 0.02

*Heterogeneity χ^2^=49.57, p<0.001, I^2^=85.87%.

**Heterogeneity χ^2^=72.95, p<0.001, I^2^=90.40%.

***Heterogeneity χ^2^=12.58, p=0.18, I^2^=28.45%.

The pooled prevalence of endophthalmitis (seven studies) was 1.0% (95% CI 0.0 to 2.0) (Table 5). Three of these studies used ECCE+PCIOL, one used a combination of different techniques and only one used SICS+PCIOL. No heterogeneity was observed between surgical techniques (p=0.87).

## Discussion

To our knowledge, this is the first systematic review and meta-analysis of VA outcomes following cataract surgery and rates of major complications in Nigeria. There is a global effort to increase cataract surgical coverage in a bid to reduce cataract-associated blindness with little emphasis on targeting improved visual outcome after cataract surgery through quality control measures.^41^

In this review, more than half (54%) of all cases received ECCE+PCIOL, only 9% had phacoemulsification surgery and IOL use was very high (94–100%). This is not surprising, as although phacoemulsification is the standard surgical technique in high-income settings, it is seldom available in low-income settings, as it requires expensive equipment and consumables, including foldable IOLs and more skilled theatre technicians and has a long learning curve for surgeons.

The mean age of patients was 54.2 y, which is younger than in similar studies from other regions, and may reflect the earlier age of onset of cataracts and shorter life expectancy in Africa. The median interval between surgery and follow-up was 12 weeks. Loss to follow-up, which was high in some studies, may reflect patient satisfaction with surgery, which will overestimate the proportion with poor outcomes.

Slightly more than half (54%) of all operated eyes achieved a good PVA (≥6/18) at ≥6 weeks after surgery and 9% had a poor outcome (VA <6/60). The proportion with good outcomes increased to 75% with best correction. This is far lower than in the UK’s National Ophthalmology Database^5^ but comparable to studies in China and southern Africa.^42–44^ Surgery on mature, hypermature and subluxated cataracts, which are common presentations in Africa, is more difficult and may have contributed to the poor visual outcomes. Late surgery could be avoided by health education for earlier cataract surgery and strengthening surgical outreach programs.

Dense cataracts would also preclude detailed preoperative posterior segment examination to detect other comorbidities, such as optic atrophy, advanced glaucoma, onchocercal chorioretinitis and diabetic retinopathy, which are more common in Nigeria than age-related macular degeneration. Earlier surgery would allow these conditions to be detected and patients counselled about the risk of poorer outcomes. The VA outcome is also lower than the WHO recommended target for BCVA of 90%, with <5% having <6/60. This may partly be due to lower or a lack of competencies of some operating surgeons, some of whom may be trainees, which may lead to more surgical complications. Competencies could be maintained or improved by continuous training of ophthalmologists and regular postoperative audits.

As in many other studies, the proportion achieving a BCVA of ≥6/18 was higher than with PVA, which may be due to surgically induced astigmatism, pre-existing refractive errors or inadequate biometry and stocks of IOLs with the powers required. PVA could be improved by better surgical techniques and biometry using newer, more accurate formulae, access to a range of IOLs of different powers and types and good stock control of IOLs.^45^

Postoperative complications such as uveitis, intraocular infection, corneal decompensation and posterior capsular opacification may also lead to poor outcomes. These may be improved by better surgical techniques, the use of viscoelastics, detailed counselling on postoperative care and prompt diagnosis and appropriate management of complications. Eight of ten operated cases were blind before surgery (VA <3/60), reflecting delayed presentation, which can be due to a wide range of well-described factors, such as a lack of awareness, distance and poverty. Late presentation has been reported in other studies, such as a prospective review of early cataract outcomes and grading.^46^ Earlier presentation requires regular eye health promotion to improve public awareness about cataract and its management.

The pooled prevalence of PCR with VL among included studies was 4.0%, which is far higher than reported in the UK’s National Ophthalmology Database^5,11^ and 3.5% reported in the USA.^47^ These complications are largely avoidable but are likely to be more frequent during surgery on subluxated or advanced cataracts where the zonules can be weak.

Endophthalmitis, was more common after ECCE surgery than other techniques. In this pooled estimate, 1.0% was reported, which is far higher than in the UK National Audit (0.03%).^11^ This may be due to the larger incision and longer operating time for ECCE than for SICS and/or poorer preoperative preparation of the eye, inadequate sterilization of surgical instruments or poor aseptic techniques, which could be prevented by advocating for a shift to SICS/phacoemulsification as well as training in astringent aseptic techniques.

The findings of this meta-analysis need to be interpreted with caution because of the heterogeneity among studies. Nevertheless, our primary aim was to obtain a ‘best estimate’ of the VA outcome, which is the most consistently used measure of the visual outcome of cataract surgery. In general, the lack of a standardized follow-up period, postoperative refraction and/or differences in the surgical techniques and IOLs used in different studies may have contributed to the variability in the outcomes. However, unless the observed heterogeneity is the result of methodologic differences among the studies, the pooled estimates should provide a reasonable estimate of the overall outcome.

Strengths of our study include the pooling of the best estimate of VA outcome across all geographical zones of Nigeria and the prevalence of visually threatening complications.

Moreover, meta-analyses cannot compensate for deficiencies in the included studies, which in this study were variable follow-up, a lack of data on the type of refractive errors and the presence of other ocular morbidities that could cause vision impairment.

We therefore recommend increasing the quality of cataract surgical services being offered, especially refresher training in modern surgical techniques for ophthalmologists who have already qualified and ensuring improved standards and surgical competency for those in training to minimise and manage surgical complications. Good preoperative preparation of patients including patient selection, good biometric measurements and IOL power determination with newer generation formulae as well as provision and use of appropriate types of IOLs will improve case selection and VA outcomes.

We also recommend instituting quality control measures and formulation of a structured protocol for postoperative management and follow-up, as well as standardization of metrics of reporting outcomes in future research to allow international comparison.

## Conclusions

We provided a detailed systematic review and meta-analysis of VA outcomes in all hospital-based cataract surgeries in Nigeria over a 28-y period. The findings indicate that VA outcomes of cataract surgery in Nigeria are far below the WHO benchmark of 90% achieving a good BCVA. Uncorrected refractive errors were an important cause of poor outcomes, as indicated by better outcomes after refraction. PCR and endophthalmitis were also higher than in other studies.

## Supplementary Material

ihaf161_Supplemental_File

## Data Availability

All the data underlying this article are available in the article and its online supplementary material.
