# Supplementary material for: Visual acuity outcome and complications of cataract surgery in Nigeria: a systematic review and meta-analysis
Source: Int Health. 2026 Mar 25;18(5):693–704. doi: 10.1093/inthealth/ihaf161 (PMC13530282; doi:10.1093/inthealth/ihaf161)
Supplement: Supplementary file 1 [file ihaf161_supplemental_file.docx]

**Methodological quality assessment**

**Supplementary Table 1:** Methodological quality assessment of included studies using modified Newcastle - Ottawa Scale (NOS)

| **Author**  **reference** |  | **Criteria** | | | | | | | | |
| --- | --- | --- | --- | --- | --- | --- | --- | --- | --- | --- |
|  |  | **Selection** | | | | **Comparability** | | **Outcome** | |  |
|  | **Study Design** | **Representativeness of the sample** | **Sample size** | **Non –responders** | **Ascertainment of exposure/risk factor** | **The study controls for the most important factor** | **The study control for any additional factor** | **Assessment of the outcome** | **Statistical test** | **Quality score** |
| Alhassan MB, et al.^25^ | Retrospective | A* | A* | B* | A* | - | - | B* | A* | 8 |
| Ezegwui RI, et al^26^ | Prospective | A* | A* | A* | A** | B* | - | B* | A* | 7 |
| Obiudu HC, et al. ^27^ | Prospective | A* | B* | A* | B* | - | - | B* | A* | 5 |
| Odugbo OP, et al.^28^ | Prospective | A* | A* | A* | A** | - | - | B* | A* | 7 |
| Olawoye OO, et al^29^ | Prospective | A* | A* | A* | A** | B* | - | B* | A* | 8 |
| Olawoye OO, et al^30^ | Retrospective | A* | A* | A* | A** | B* | - | B* | A* | 8 |
| Oladigbolu KK, et al.^31^ | Prospective | A* | B* | B* | B* | - | - | B* | A* | 5 |
| Ejimadu CS, et al^32^ | Prospective | A* | A* | C* | B* | B* | - | B* | A* | 5 |
| Nkanga DG, et al.^33^ | Prospective | A* | A* | C* | B* | B* | - | B* | A* | 6 |
| Shu'aibu A, ^34^ | Comparative | A* | B* | A* | B* | - | B* | B* | A* | 5 |
| Oderinlo O et al^35^ | Retrospective | A* | A* | A* | A** | B* | - | B* | A* | 9 |
| Fasina O, et al^36^ | Retrospective | A* | A* | A* | A** | B* | - | B* | A* | 9 |
| Oladigbolu KK^37^ | Prospective | A* | A* | C* | B* | B* | - | B* | A* | 7 |
| Bulus SS, et al.^38^ | Prospective | A* | A* | A* | A** | B* | - | B* | A* | 8 |
| Bogunjoko TJ^49^ | Retrospective | A* | A* | A* | A* | B* |  |  |  | 6 |
| Jeneral AR^40^ | Retrospective | A* | A* | B* | A* | - | - | B | A | 5 |

*Note: from each item account point. (Accept the study for each study design based on the total score of ≥5)*

Selection: (Maximum 5 stars)
1) Representativeness of the sample: A*) Truly representative of the average in the target population(all subjects or random sampling) .B*) Somewhat representative of the average in the target population. * (nonrandom sampling) .C*) Selected group of users. D*) No description of the sampling strategy.
2) Sample size: A*) Justified and satisfactory. B*) Not justified.
3) Non-respondents: A*) Comparability between respondents’ and non-respondents characteristics is
established, and the response rate is satisfactory. B* ) The response rate is unsatisfactory, or the comparability between respondents
and non-respondents is unsatisfactory. C*) No description of the response rate or the characteristics of the responders and
the non-responders.
4) Ascertainment of the exposure (risk factor): A) validated measurement tool. ** .B) Non-validated measurement tool, but the tool is available or described. C*) No description of the measurement tool.
Comparability: (Maximum 2 stars)
1) The subjects in different outcome groups are comparable, based on the study design or analysis. Confounding factors are controlled. A*) The study controls for the most important factor (select one). B* ) The study control for any additional factor. *
Outcome: (Maximum 3 stars)
1) Assessment of the outcome: A) Independent blind assessment. **,B) Record linkage. **,C) Self report. *,D) No description.
2) Statistical test: a) The statistical test used to analyze the data is clearly described and appropriate, and the measurement of the association is presented, including confidence intervals and the probability level (p value). *,B) The statistical test is not appropriate, not described or incomplete

**Supplementary Table 2:** The risk of bias assessment tool for the included studies.

| **Author**  **[reference]** | Representation | Sampling | Random selection | Non-response bias | Data collected | Case definition | Reliability &validity of tool | Mode of data collection | Length prevalence period | Numerator & denominator | **The overall risk of bias** |
| --- | --- | --- | --- | --- | --- | --- | --- | --- | --- | --- | --- |
| Alhassan MB, et al.^25^ | Yes | Yes | No | Yes | Yes | Yes | Yes | Yes | No | Yes | Low risk |
| Ezegwui RI, et al^26^ | Yes | Yes | No | Yes | Yes | Yes | Yes | Yes | Yes | Yes | Low risk |
| Obiudu HC, et al. ^27^ | Yes | Yes | No | Yes | Yes | Yes | Yes | Yes | Yes | Yes | Low risk |
| Odugbo OP, et al.^28^ | Yes | Yes | No | Yes | Yes | Yes | Yes | Yes | Yes | Yes | Low risk |
| Olawoye OO, et a29 | Yes | Yes | No | Yes | Yes | Yes | Yes | Yes | Yes | Yes | Low risk |
| Olawoye OO, et al^30^ | Yes | Yes | No | Yes | Yes | Yes | Yes | Yes | Yes | Yes | Low risk |
| Oladigbolu KK, et al.^31^ | Yes | Yes | No | Yes | Yes | Yes | Yes | Yes | Yes | Yes | Low risk |
| Ejimadu CS, et al^32^ | Yes | Yes | No | Yes | Yes | Yes | Yes | Yes | No | Yes | Low risk |
| Nkanga DG, et al.^33^ | Yes | Yes | No | Yes | Yes | Yes | Yes | Yes | Yes | No | Low risk |
| Shu'aibu A. ^34^ | Yes | Yes | No | Yes | Yes | Yes | Yes | Yes | Yes | Yes | Low risk |
| Oderinlo O et al^35^ |  |  | No | Yes | Yes | Yes | Yes | Yes |  | Yes | Low risk |
| Fasina O, et al^36^ | Yes | Yes | No | Yes | Yes | Yes | Yes | Yes | Yes | Yes | Low risk |
| Oladigbolu KK^37^ | Yes | Yes | No | Yes | Yes | Yes | Yes | Yes | Yes | Yes | Low risk |
| Bulus SS et al^38^ | Yes | Yes | No | Yes | Yes | Yes | Yes | Yes | Yes | Yes | Low risk |
| Bogunjoko TJ et al^39^ | Yes | Yes | No | Yes | Yes | Yes | Yes | Yes | Yes | Yes | Low risk |
| Jeneral AR^40^ | Yes | Yes | No | Yes | Yes | Yes | Yes | Yes | Yes | Yes | Low risk |

- **Note**

Risk of bias assessment tool: Yes (low risk); No (high risk)

1. Representation: Was the study population a close representation of the national population?

2. Sampling: Was the sampling frame a true or close representation of the target population?

3. Random selection: Was some form of random selection used to select the sample OR was a census undertaken?

4. Non-response bias: Was the likelihood of non-response bias minimal?

5. Data collection: Were data collected directly from the subjects?

6. Case definition: Was an acceptable case definition used in the study?

7. Reliability and validity of study tool: Was the study instrument that measured the parameter of interest show to have reliability and validity?

8. Data collection: Was the same mode of data collection used for all subjects?

9. Prevalence period: Was the length of the prevalence period for the parameter of interest appropriate?

10. Numerators and denominators: Were the numerator(s) and denominator(s) for the parameter of interest appropriate?

The overall risk of bias scored based on the number of high risk of bias per study: low risk (≥8), moderate risk (5–7), and high risk (≤4)
